# Efficient attention-based CNN network (EANet) for multi-class maize crop disease classification

**DOI:** 10.3389/fpls.2022.1003152

**Published:** 2022-10-12

**Authors:** Saleh Albahli, Momina Masood

**Affiliations:** ^1^ Department of Information Technology, College of Computer, Qassim University, Buraydah, Saudi Arabia; ^2^ Department of Computer Science, University of Engineering and Technology, Taxila, Pakistan

**Keywords:** maize crop disease, deep-learning, attention mechanism, convolutional neural network, image classification

## Abstract

Maize leaf disease significantly reduces the quality and overall crop yield. Therefore, it is crucial to monitor and diagnose illnesses during the growth season to take necessary actions. However, accurate identification is challenging to achieve as the existing automated methods are computationally complex or perform well on images with a simple background. Whereas, the realistic field conditions include a lot of background noise that makes this task difficult. In this study, we presented an end-to-end learning CNN architecture, Efficient Attention Network (EANet) based on the EfficientNetv2 model to identify multi-class maize crop diseases. To further enhance the capacity of the feature representation, we introduced a spatial-channel attention mechanism to focus on affected locations and help the detection network accurately recognize multiple diseases. We trained the EANet model using focal loss to overcome class-imbalanced data issues and transfer learning to enhance network generalization. We evaluated the presented approach on the publically available datasets having samples captured under various challenging environmental conditions such as varying background, non-uniform light, and chrominance variances. Our approach showed an overall accuracy of 99.89% for the categorization of various maize crop diseases. The experimental and visual findings reveal that our model shows improved performance compared to conventional CNNs, and the attention mechanism properly accentuates the disease-relevant information by ignoring the background noise.

## 1 Introduction

Maize is one of the most essential cereal crops, having the largest production worldwide that can be farmed in a variety of climates. It is highly valued for its widespread usage as a staple diet for humans and high-quality feed for animals. Furthermore, maize is the principal raw material for a wide range of industrial goods. Regardless of its high grain yield potential, the sensitivity of maize crops to various diseases is a significant barrier to increasing yields and results in a 6-10% annual loss in production (Zhang et al., 2021). As a result, timely detection and monitoring of maize diseases are critical during the growing season to control their spread. The accurate identification of the diseases is strongly dependent on the availability of domain specialists and plant pathologists, as well as requires good observation skills and knowledge of specific disease signs. Moreover, the manual identification process consumes huge resources and time since it requires continual plant monitoring, which is costly when working with large farms. Thus, rapid and precise methods of recognizing maize diseases are needed to monitor the crop and take prompt action to cure the infections.

Currently, computer vision (CV) technology and machine learning (ML)-based methods are progressively applied to the field of plant disease identification due to their expert-level performance in challenging situations (Vishnoi et al., 2021). As a result, a digital image-based automatic disease diagnosis strategy in the maize crop is a feasible and viable alternative to the manual inspection process. Traditional image processing techniques include gray level co-occurrence matrix (GLCM) (Kaur et al., 2018), scale-invariant feature transform (SIFT) (Chouhan et al., 2021), local binary patterns (LBP) (Pantazi et al., 2019), and histogram of oriented gradient (HOG) (Wani et al., 2021), etc., are widely adopted for purpose of identifying plant diseases. These methods extract different attributes (e.g., shape, texture, and color) and statistical traits to characterize the attributes of diseased spots in affected leaf images (Thakur et al., 2022). The extracted hand-crafted features are classified using conventional ML algorithms primarily the Naive Bayes (NB) Classifier (Panigrahi et al., 2020; Mohapatra et al., 2021), support vector machine (SVM) (Chung et al., 2016), K-Nearest Neighbor (KNN) algorithm (Hossain et al., 2019), and artificial neural network (ANN) (Patil et al., 2017) for categorizing leaf diseases. In a study on the detection of maize diseases, the authors compared different ML methods including NB, KNN, SVM, Decision Tree (DT), and Random Forest (RF) (Panigrahi et al., 2020). Aravind et al. (2018) extracted textural characteristics of maize leaf disease using the GLCM and subsequently classified maize illnesses using multi-class SVM. However, the overall performance of traditional ML methods is primarily constrained by feature extraction and representation approaches.

Recently, deep learning (DL)-based methods have achieved tremendous improvement in identifying plant diseases (Lee et al., 2017; Hasan et al., 2020; Lee et al., 2020a; Albattah et al., 2022). DL techniques can automatically discover the representations necessary to perform classification. Convolutional neural network (CNN), a special type of DL architecture, has shown remarkable performance in several areas including agriculture (Lee et al., 2015; Albattah et al., 2022), medical imaging (Nawaz et al., 2021; Masood et al., 2021), fake news detection (Saleh et al., 2021), etc. CNNs are capable of extracting discriminative features from input samples and effectively perform visual recognition tasks (Lee et al., 2015). The CNN structure can automatically learn key properties from the training data without any human supervision. The CNNs are extensively applied for the categorization of various plant leaf diseases (Sethy et al., 2020; Ngugi et al., 2021; Albattah et al., 2022). In a large number of studies, researchers fine-tuned pre-built CNN models such as AlexNet (Rangarajan et al., 2018), GoogleNet (Mohanty et al., 2016), ResNet (Subramanian et al., 2022), InceptionNet (Haque et al., 2022), Efficientnet (Liu et al., 2020) and DenseNet (Waheed et al., 2020; Baldota et al., 2021) employing transfer learning for leaf disease identification. Some of the studies have suggested novel CNN architecture for plant disease identification (Picon et al., 2019; Agarwal et al., 2020; Zhang et al., 2021; Xiang et al., 2021). Although, the studied CNN approaches in these works have shown effective performance and appear to learn disease-specific feature representations, however, their performance is affected by background noise (Hasan et al., 2020; Subramanian et al., 2022). In (Atabay, 2017), the authors trained a CNN model for the identification of tomato plant diseases and analyzed that the model has neuron activations predominantly in the image background, rather than the diseased region. This suggests that a CNN model is more likely to learn irrelevant features other than the visual representation of plant disease. Furthermore, it has been demonstrated that background suppression using image segmentation techniques does not result in better generalization outcomes for disease identification in a real environment (Mohanty et al., 2016). Simultaneously, to deal with the influence of numerous visual disturbances such as non-uniform lightning conditions, distortion, and blur, it is required to improve CNN performance in order to tackle fine-grained plant disease identification tasks (Lee et al., 2020b). At present, object identification-based algorithms are now being developed and used for the localization and categorization of plant diseases. Region-based CNN can better localize diseased areas in the presence of complicated background settings, however, it involves labor-intensive annotations of disease locations (Zhou et al., 2019; Albattah et al., 2022; He et al., 2022).

Recent studies show that the attention method can be supplemented with CNN to obtain discriminative features of the region of interest (Guo et al., 2022). The attention mechanism enables a CNN network to use the global information of features, focusing on the most important characteristics while suppressing less informative data, hence increasing the efficacy of a network’s feature representation. These methods are effectively applied in the field of CV and achieved good results (Guo et al., 2022). Particularly for crop leaf disease detection, identifying and focusing on disease-affected areas is critical for attaining high classification accuracy (Zeng and Li, 2020; Yang et al., 2020; Zhu et al., 2021). Limited studies have investigated attention techniques for the precise categorization of maize leaf disease (Chen et al., 2021; Zeng et al., 2022a; Qian et al., 2022).

Despite tremendous improvements, there is still a need for improvement in diagnosing and classifying maize leaf disease in actual field situations. For instance, even though certain models provide exceptionally high accuracy on maize datasets created in a lab setting, they frequently produce unsatisfactory identification results in the real world (Ahila Priyadharshini et al., 2019; Baldota et al., 2021; Subramanian et al., 2022). This is due to insufficient disease feature extraction, which results in a lack of critical disease information. The key challenges in accurately classifying the maize disease are the high degree of visual similarity between categories, the extensive background noise in the field environment, and the inconsistent placement of various crop diseases (He et al., 2022; Qian et al., 2022).

These observations motivated us to investigate novel methods for identifying maize diseases that can automatically learn the robust representations of interest in the input image that corresponds to unhealthy portions and subsequently identify the disease. In this work, we proposed an efficient and effective method by incorporating an attention mechanism in the EfficientNetv2 CNN (Tan and Le, 2021) architecture namely EANet for fine-grained maize crop disease identification. The proposed EANet model effectively computes high-level representations and categorizes them in their respective class using an end-to-end training method. Furthermore, the attention mechanism enhances the learning ability of the CNN by providing fine details of the salient characteristics such as disease portions (Zhu et al., 2021). The following are the major contributions:

*We proposed EANet, a lightweight CNN model that extracts robust and discriminative features of interest and thus achieves high accuracy for fine-grained categorization of different maize crop diseases while having less computational complexity.
*We incorporated the spatial and channel attention method into the CNN architecture which enhances its capacity of learning the inter-channel connections and space-wise position attributes to improve the identification of maize leaf diseases in real environment settings.
*To prevent the model overfitting and deal with class imbalance data, we employed transfer learning and multi-class focal loss which boosts the maize disease classification accuracy.
*To show the efficacy of the proposed EANet model, we conducted extensive comparative experiments to analyze the performance using a maize disease image database collected from three different online available sources. The proposed technique effectively classifies the maize leaf diseases in the presence of complex environmental situations, such as blurring, noise, nonuniform lightning conditions, and variation in the color, size, and location of disease spots.

The remaining paper is arranged as follows. Section 2 presents the summary of prior research presented to categorize maize leaf diseases from digital images. Section 3 presents material and methods mentioning the details of the dataset used for experimentation and an explanation of the proposed maize leaf disease classification model with its architectural details. Section 4 describes implementation details. It also discusses details of the different experiments performed and their outcomes. Finally, section 5 concludes our research and presents some future directions.

## 2 Related work

Researchers have presented various approaches to categorize, identify, and extract the characteristics of plant diseases. DL, as well as image processing and classical ML techniques, have been widely adopted in the agriculture domain to accomplish this. In this section, we have presented an overview of some of the existing works that have been developed to classify corn leaf diseases from digital images. The existing literature is broadly divided into two major categories i.e., traditional ML and DL-based approaches. The ML-based methods use algorithms to extract hand-crafted features and a classifier to perform categorization. In (Aravind et al., 2018), the authors computed textural features using a histogram and a GLCM and trained multi-class SVM to perform the categorization of three maize diseases such as Common Rust (CR), leaf blight, and cercospora leaf spot. Zhang et al. (2015) developed a method that employed a genetic algorithm to automatically determine the kernel function and penalty factor in SVM. This approach reported an overall classification accuracy of 90.25%. Ikorasaki et al. (Ikorasaki and Akbar, 2018) suggested the bayesian theorem to construct an expert diagnostic system for the identification of maize crop disease based on the symptoms, with a precision rate of 90%. Zhang et al. (2015) suggested a method that first segments the diseased region and then computes a feature vector based on the shape, color, and texture aspects of the segmented region. Then, the KNN classification technique was used to categorize these features into five different maize diseases and attained an overall identification accuracy of 90.30%. Xu et al. (2015) suggested an adaptive weighting multi-classifier fusion approach for identifying maize leaf disease. This approach was used to test seven prevalent types of maize leaf disease. The average rate of recognition was 94.71%. In (Zhang and Yang, 2014), the authors employed the SVM technique to categorize images of maize disease acquired from the internet, with an overall recognition accuracy of 83.2%. Qi et al. (2016) presented an image processing-based method for the categorization of maize leaf disease images. Initially, the retinex algorithm was employed to improve the image. Then, an automated threshold approach in R-G gray space was used to extract disease spots, color, texture, and invariant moments. The principal component analysis approach was employed to obtain dominant features and the SVM was used to identify three common maize diseases such as CR, Southern Leaf Blight (SLB), and curvularia lunata. The overall recognition accuracy obtained was 90.74%. However, the classification accuracy of these ML-based methods for various maize leaf diseases is low due to less discriminative power of extracted hand-crafted features.

Several DL-based methods have been widely adopted for the classification of maize leaf disease due to their improved feature extraction and representation capabilities. Haque et al. (2022) developed an Inceptionv3-based architecture for the classification of healthy maize leaves from diseased ones. Initially, several augmentation strategies such as flipping, rotating, skew, and distortion was applied to enhance the diversity of input data. Then, an Inceptionv3 model with a global average pooling layer was used to compute the keypoint vector and perform classification. In the work by Subramanian et al. (2022), DL models such as ResNet50, InceptionV3, VGG16, and Xception were evaluated to recognize maize leaf diseases. They conducted transfer learning and bayesian hyperparameter optimization to enhance the performance. The Xception network showed the highest recognition accuracy among others, however, it involves a larger number of parameters and is computationally complex. In (Ahila Priyadharshini et al., 2019), the authors suggested a modified LeNet CNN architecture with a smaller kernel size for disease categorization in maize leaves. This method achieved an accuracy of 97.89% with transfer learning. Similarly, in another study (Baldota et al., 2021) DenseNet121 model was employed. The model attained an accuracy of 98.45% on maize disease samples from the PlantVillage database and 91.49% under real-environment conditions such as varying lighting and jitter. In (Liu et al., 2020), the authors used the EfficientNet-b0 CNN model to classify several maize leaf diseases and adopted transfer learning to accelerate the training. This method showed improved recognition accuracy; however, requires extensive performance evaluation on a challenging database having noisy samples or real-environment complexities. Zhan et al. (Zhang et al., 2018) introduced an improved GoogLeNet and Vgg CNN for the classification of nine different maize diseases. The authors explored different pooling layer combinations, activation functions, and dropout operations to decrease the number of model parameters. This method obtained an average accuracy of 98.9%; however, the method is evaluated on a database having limited diversity. Lv et al. (2020) proposed a CNN architecture namely DMS-Robust Alexnet with dilated and multiscale convolution for the classification of maize crop disease. Initially, the input images were enhanced using preprocessing, and then, data augmentation was employed to increase the size of the input database. The average recognition accuracy for this method was 98.62%. In (Zhang et al., 2021), the authors introduced a multi-activation function (MAF) module based on a combination of various activation methods (Sigmoid, ReLU, Mish, Tanh, and LeakyReLU) in the CNN model to enhance the performance of maize leaf disease identification. Initially, numerous image pre-processing algorithms, such as DCGAN, were utilized to extend and enrich the data of diseased samples. Then, various baseline CNN models such as AlexNet, VGG19, ResNet50, DenseNet161 and were evaluated by integrating the MAF module. This approach showed the highest prediction accuracy of 97.41% using ResNet50; however, the performance is limited over the noisy samples. In (Amin et al., 2022), the authors developed a model using the EfficientNetB0, and DenseNet121 network to compute deep keypoints for the categorization of maize leaf disease. They fused the extracted features to obtain a more descriptive representation before performing classification. This method is evaluated using corn leaf disease samples from the PlanVillage database and thus has limited generalization for real environment conditions. Zeng et al. (2022) presented a CNN model namely SKPSNet-50 for the recognition of different maize leaf diseases at an early stage. A selected kernel unit with a swish activation function was integrated into the ResNet50 model to improve the feature extraction. This approach showed an overall accuracy of 92.9% for categorizing six different maize diseases. Li et al. (2022) presented an improved one-stage detection model i.e., YOLOv5 using multi-scale feature fusion for the detection of corn leaf infections. A spatial pyramid pooling and coordinate attention mechanism were introduced in the backbone network to improve the feature extraction and classification performance. This approach shows an improved generalization performance, however, the accuracy decreases for small disease target localization. In (Pan et al., 2022), the authors evaluated different CNN models such as VGG16, VGG19, AlexNet, and GoogleNet using different loss functions Softmax, ArcFace, and CosFace for the identification of Northern Corn Leaf Blight (NLB) disease. This method showed the highest accuracy of 99.94% using GoogleNet with Softmax loss function. Singh et al. (2022) employed transfer learning to train the AlexNet CNN for the identification of maize leaf disease. The method is evaluated using the PlantVillage database and attained an accuracy of 99.16% on 100 epochs. However, the use of maize leaf disease images with a simple background in CNN models limit the practical usefulness of such models.

The attention mechanism is an effective supplementary method for improving traditional feature extraction. In (Zeng et al., 2022a), the authors proposed a lightweight dense-scale network (LDSNet) that combined dense dilated convolutional blocks and a coordinated attention fusion mechanism for the identification of maize diseases. The dilated convolutional layers improved the model receptive field and provided computation of disease features at different scales. The overall identification accuracy for maize leaf diseases and healthy leaves was 95.4%. Yin et al. (2022) introduced an improved GoogleNet architecture for the grading of maize SLB spots. A dilated inception block was added to improve the extraction of multi-scale features. The channel attention mechanism was then integrated to emphasize the relevance of inter-channel correlations for input features. This approach attained an accuracy of 97.12% on a self-created database. Chen et al. (2021) presented a framework namely Mobile-DANet to categorize maize crop diseases. The architecture comprises a DenseNet-based CNN having depthwise separable convolution and spatial-channel attention blocks. This approach showed an average accuracy of 98.50% on plantVillage and 95.86% on database samples having noisy background conditions. Qian et al. (2022) suggested an approach based on a vision transformer for the identification of maize leaf disease. Initially, a CNN network was used to extract the feature vector and encode it into a token matrix. Then, a multi-head self-attention was introduced in the transformer encoder to compute the correlation between tokens. This method shows improved classification accuracy; however, the performance is limited by the token representation dimension resulting in the loss of semantic information between neighboring patches. He et al. (2022) presented the MFasterR-CNN model for the detection and categorization of maize crop diseases. A VGG16-based backbone network was used for the extraction of features in the Faster-RCNN network. This method showed improved recognition accuracy, however, it employs a selective search algorithm for the detection of infected regions which is slow. Moreover, it requires annotated data which is an expensive process.

The comparison of the existing techniques for the categorization of maize leaf disease is presented in **Table 1**. According to the reviewed literature, a number of works have been attempted to perform maize disease identification and classification using CNN models. It can be observed that maize disease classification accuracy has significantly improved. However, these methods show robust performance for maize disease classification utilizing samples with a simple background or surroundings. The performance of existing techniques is vulnerable to environmental effects and degrades on images with complicated backgrounds having numerous visual disturbances such as non-uniform lightning conditions, distortion, and blur (**Table 1**). These factors limit the practical applicability of existing models for the classification of multiple maize leaf diseases. As a result, there is still room for improvement in approaches in terms of generalization, computational, and processing time complexities.

**Table 1:** Comparison of existing techniques.

Reference	Year	Method	Dataset	Limitations	Performance (Accuracy)
Zhang et al. (2015)	2015	Genetic algorithm-based SVM	No details given	This method involves extensive pre-processing and manual feature extraction and thus, has low generalizability to unseen cases.	90.25%
Xu et al. (2015)	2015	Multiple features such as color co-occurrence matrix, color moment, and LBP with adaptive weighting multi-classifier fusion	Private self-created	This approach is quite complicated and has increased recognition time due to the fusion process.	94.71%
Qi et al. (2016)	2016	Textural features with principal component analysis and SVM	No details given	This approach lacks generalization.	90.74%
Aravind et al. (2018)	2018	Textural features using GLCM and Histogram, multi-class SVM	PlantVillage	This approach requires the manual selection and the extraction of features and thus has a low generalization to variations in blurring, noise, non-uniform lightning conditions.	83.7%
Zhang et al. (2018)	2018	Improved GoogLeNet and VGG CNN	PlantVillage and google website	This method lacks robustness and utilized a small dataset having 500 total samples.	98.9%
Ahila Priyadharshini et al. (2019)	2019	Modified LeNet CNN	PlantVillage	This approach involves extensive preprocessing and lacks analysis of model robustness.	97.89%
Liu et al. (2020)	2020	EfficientNet-B0	Crop disease global AI-challenge	This work lacks detailed experimental results and analysis.	98.52%
Lv et al. (2020)	2020	AlexNet with dilated and multiscale convolution	PlantVillage, crop disease global AI-challenge 2018 and google website	This model involves a large number of parameters and is computationally complex.	98.62%
Baldota et al. (2021)	2021	DenseNet121	PlantVillage	This approach lack robustness on noisy data	91.49%
Haque et al. (2022)	2022	Inception-v3	PlantVillage	Lacks analysis of model robustness on real environment samples.	95.99%
Subramanian et al. (2022)	2022	VGG16, ResNet50, InceptionV3, and Xception with Bayesian for hyperparameter optimization	PlantVillage	The work lacks model robustness analysis using real-world instances.	99%
He et al. (2022)	2022	Two-stage object detection model MFaster-RCNN with VGG16-based feature extraction	Private self-created	This approach is computationally complex and involves manual annotation of samples for training.	97.23%
Amin et al. (2022)	2022	Deep feature fusion using EfficientNetB0, and DenseNet121	PlantVillage	The results are reported using a dataset captured in a controlled environment.	98.56%
Zeng et al. (2022)	2022	Modified ResNet50-based model i.e., SKPSNet-50	Private self-created	The dataset contained only 1452 total samples and the approach suffers from the issue of over-fitting.	92.9%
Li et al. (2022)	2022	One-stage object detection YOLOv5 with multi-scale feature fusion network for feature extraction	Kaggle-Corn leaf infection dataset	This approach requires annotated samples for model training.	71.01% (Sensitivity)
Pan et al. (2022)	2022	Multiple feature extractors such as VGG16, VGG19, AlexNet, and GoogleNet using different loss functions Softmax, ArcFace, and CosFace	Private self-created	This study is limited to binary classification.	99.94%
Singh et al. (2022)	2022	AlexNet CNN	Private self-created	This approach involves a large number of parameters, longer training time i.e., 100 epochs and is computationally complex.	99.16%

## 3 Materials and methods

In this section, we explained the dataset and the method adopted for the maize leaf disease identification. We have discussed the proposed EANet architecture and its details for fine-grained various maize disease classification task.

### 3.1 Dataset

**Figure 1:**
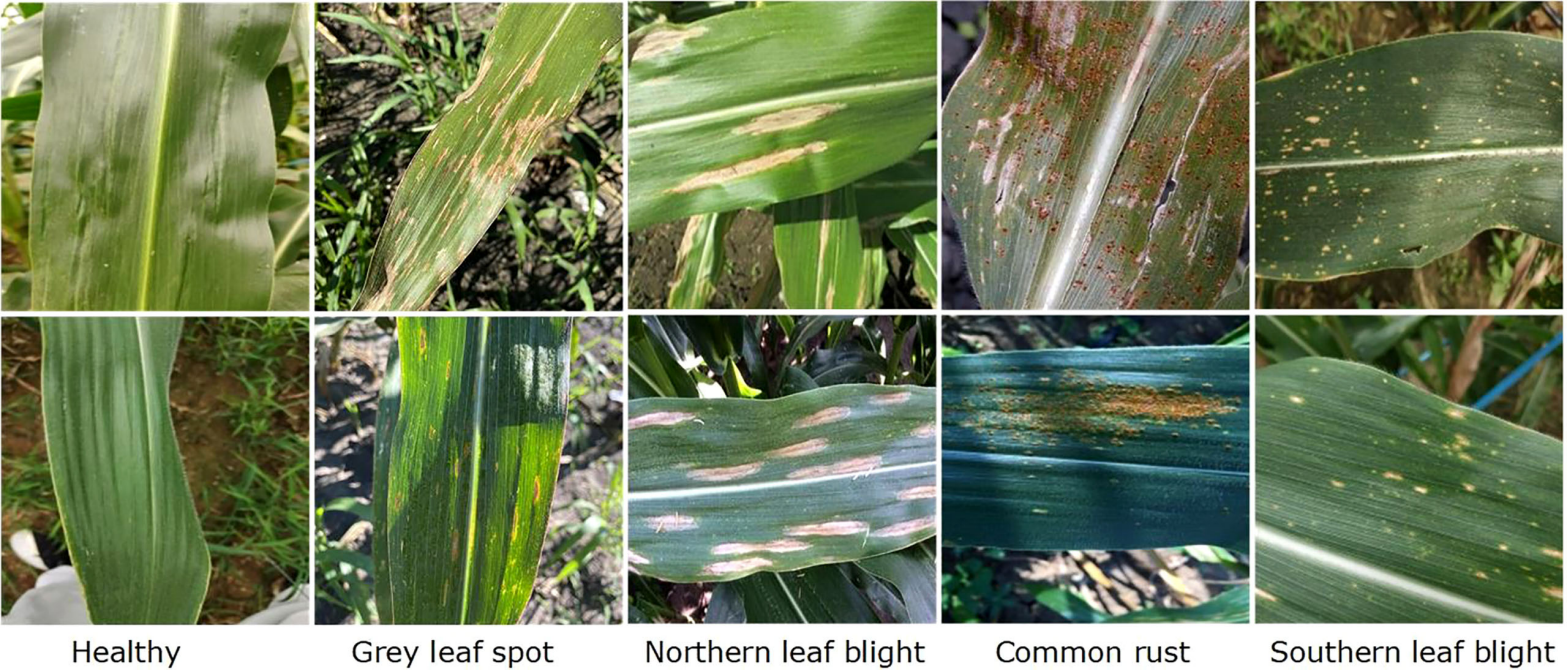
Sample images showing different types of maize leaf diseases.

**Figure 2:**
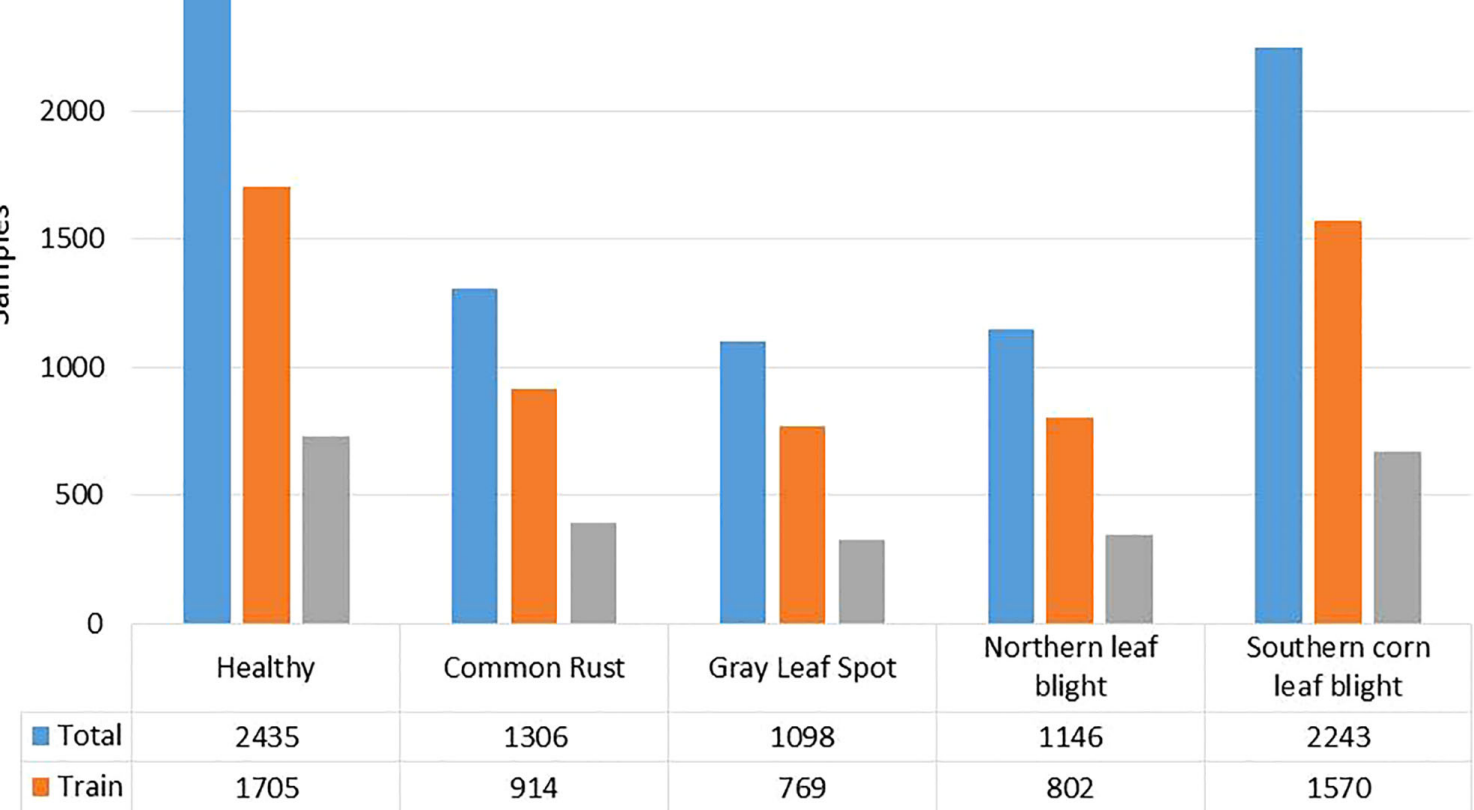
Number of class-wise samples in the maize leaf disease dataset used. To improve the diversity of the images and prevent over-fitting problems during training, data augmentation methods such as random angle rotation, flipping, horizontal or vertical translation, scale alteration, and color jittering were applied. With this approach, there were at least 2500 samples in each group. The images were resized to a dimension of 240 × 240 pixels. **Figure 3** displays some instances of augmented images.

**Figure 3:**
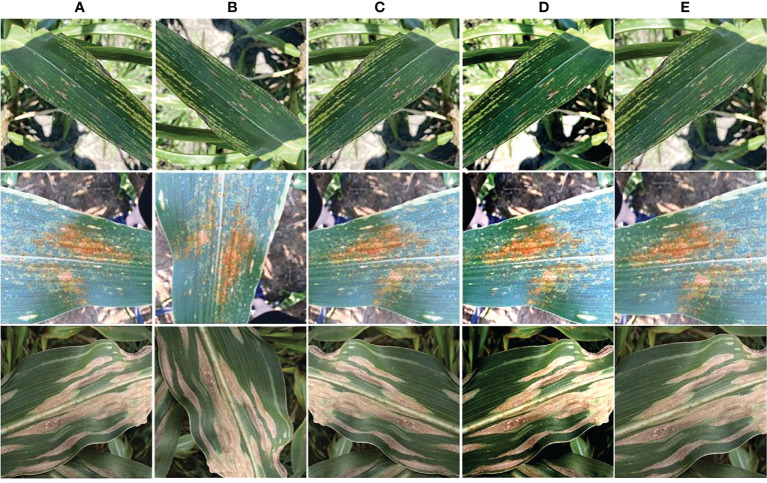
Augmented images **(A)** original sample, **(B)** angle rotation, **(C)** flipping, **(D)** color change, and **(E)** scaling.

In this work, we have used the maize leaf disease images from three different online available data sources to assess the classification effectiveness of our technique. To show the generalization of the proposed method, we have used the databases having the samples captured in both a controlled and real environment. The Maize Disease dataset (Corn or maize leaf disease dataset, 2022) comprised images from the PlantVillage and PlantDoc databases. It consists of a total of 4188 samples having three maize leaf diseases: 1306 images of CR, 574 images of grey leaf spot (GLS), 1146 images of northern corn leaf blight (NLB), and 1162 images of healthy maize leaf. This database (Corn or maize leaf disease dataset, 2022) contains images of leaves taken in various orientations and under controlled background settings with approximately even illumination levels. The other database used in our study is presented in (Qian et al., 2022). The database contains 1273 samples of healthy leaves, 1023 of CR, and 2243 images of SLB disease. The samples were recorded in the natural environment using mobile phones under normal and uncontrolled lighting settings. The number of samples with GLS disease was lower than the other categories. A total of 524 images of GLS disease captured in real-environment were taken from the source (Ahmad et al., 2021). Overall, the dataset we utilized to categorize maize plant leaves is complex in nature as it contains samples with different diseases captured in real environment settings. Moreover, it contains samples having disease regions of various sizes, colors, and shapes, as well as image distortions such as noise, lighting, and blurring. **Figure 1** shows a few samples from the database, while **Figure 2** provides a class-wise partition of the dataset. To improve the diversity of the images and prevent over-fitting problems during training, data augmentation methods such as random angle rotation, flipping, horizontal or vertical translation, scale alteration, and color jittering were applied. With this approach, there were at least 2500 samples in each group. The images were resized to a dimension of 240 × 240 pixels. **Figure 3** displays some instances of augmented images.

### 3.2 Proposed EANet model

To accurately identify different maize leaf diseases, we introduced EANet, a lightweight CNN model built by using the Efficinetnetv2 CNN (Tan and Le, 2021) model with an attention mechanism. The proposed EANet model extracts effective representations by using an attention mechanism that allows to focus on disease regions and enhances the ability for fine-grained classification of maize diseases. The crucial information of maize disease is the leaf area where the infectious spots are located. The color and texture properties of these local regions serve as the feature information from a visual perspective. Usually, the high-level features extracted by CNN may contain redundant background information that interferes with the representation of infectious spots. To alleviate this, we used the Efficinetnetv2 CNN that extracts more robust features of the image and downsamples into a lower dimension without losing its characteristics. These features are used by the attention module that improves local related features and restricts irrelevant features at spatial and channel levels (Woo et al., 2018; Zhu et al., 2021).

**Figure 4:**
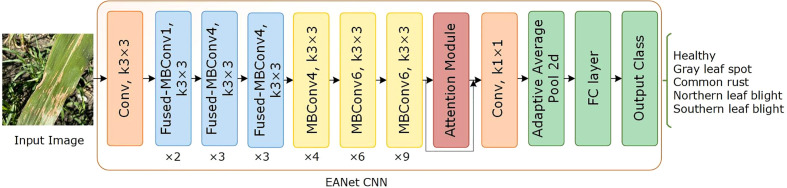
Architecture of proposed EANet model for the classification of maize leaf disease.

The network architecture of the proposed EANet approach is illustrated in **Figure 4**. It comprises of an Efficientnetv2 CNN, a spatial-channel attention module, an adaptive average pooling (AvgP) layer, and finally a classification layer. Initially, the image is sized to 240 × 240 before passing it as input to the network model. Then, an efficientnetv2-based CNN network extracts high-level feature information of the image. The attention module enhances the capacity of the model to extract disease characteristics, which increases identification accuracy. The spatial attention module uses the multiplied features from the convolutional layer and the channel attention module to compute the location of the relevant keypoints in the image. Finally, the adaptive average pooling layer learns the dependencies between several channels adaptively and alters the feature map to 1 × 1 × 1280. The fully connected (FC) layer categorizes the computed keypoints using softmax classification.

#### 3.2.1 EfficientNetv2 CNN model

The EfficientNetV2 is an enhanced variant of the EfficientNet CNNs (Tan and Le, 2019), designed to optimally use available resources while maintaining high accuracy (Tan and Le, 2021). The EfficientNet CNN architecture is designed by using a compound scaling approach that enables a baseline CNN to expand equally in three dimensions such as depth, width, and input size. Furthermore, EfficientNet models are substantially less in size when compared to other CNN models and significantly outperform on the ImageNet database (Huang et al., 2017).

**Figure 5:**
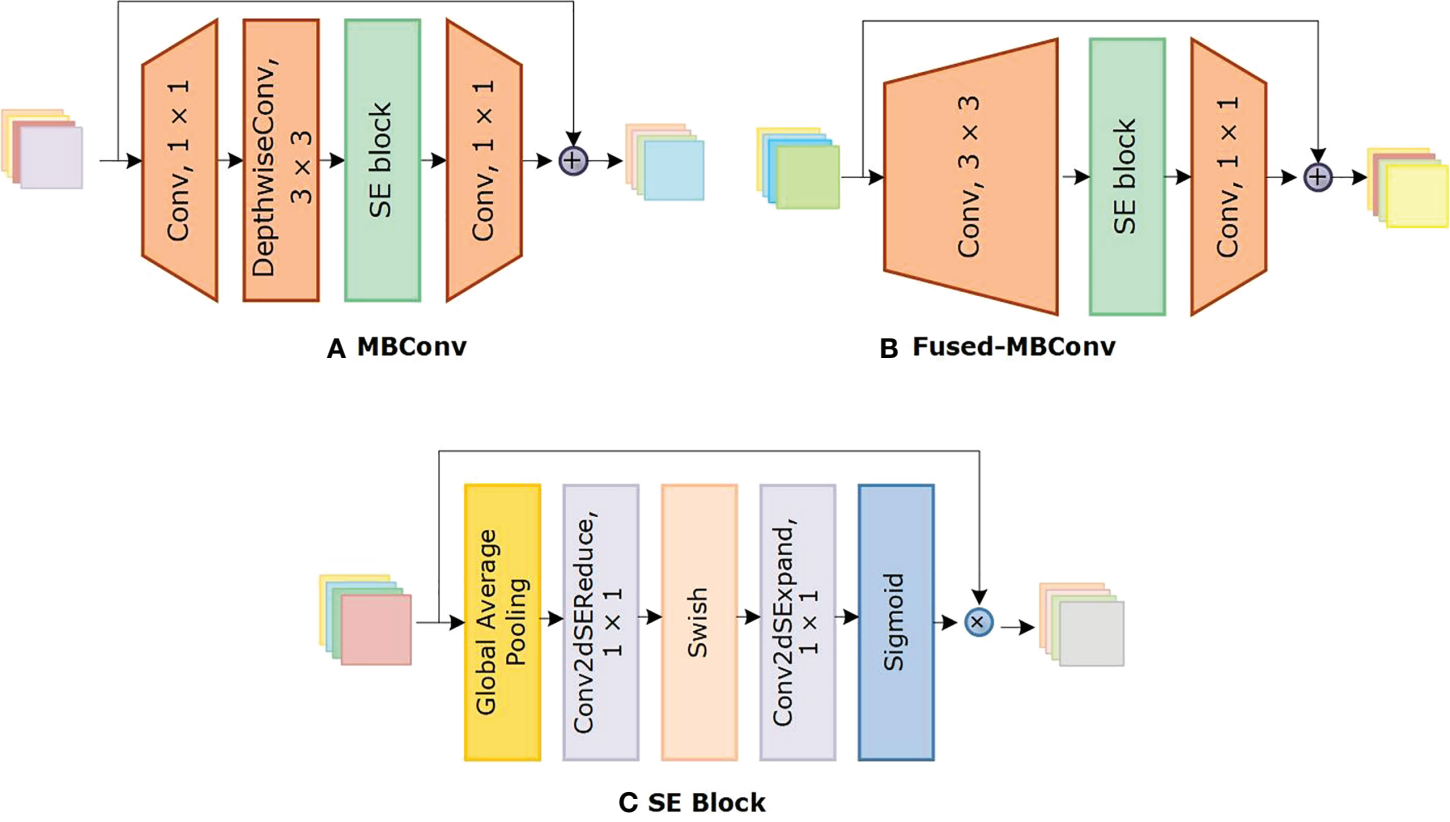
Structure of MBConv, Fused MBConv, and the SE block.

We chose the Efficientnetv2 CNN for maize leaf disease identification because of its lightweight architecture, faster training, and inference speed. **Table 2** shows the architecture of Efficientnetv2 CNN. The network mainly comprises of MBConv and Fused-MBConv blocks, which uses squeeze and excitation (SE) optimization to construct channel-wise attention and enhance the network’s feature expressiveness. **Figure 5** shows the architecture of MBConv, Fused-MBConv, and the SE block. In Efficientnetv2, the incorporation of Fused-MBConv blocks at an earlier level leads to greater parameter efficiency and faster training as compared to Efficientnetv1 (Tan and Le, 2021). The MBConv block begins with a 1×1 convolutional layer and a depthwise convolution with a 3×3 kernel size. In the Fused-MBConv block, the depthwise 3×3 convolution and expansion 1×1 convolution layers in MBConv are replaced with conventional 3×3 convolution layers. A 1×1 pointwise convolution is applied after the SE block in both MBConv, and fused-MBConv blocks to adjust the channel dimensions. Finally, a drop connection is performed, followed by a skip connection of the input.

**Table 2:** Architecture of Efficientnetv2 baseline network.

Blocks *(i)*	Layer (*f_i_*)	Resolution *(H_i_×W_i_)*	Channel *(C_i_)*	Number of layers
1	Convolution layer [3 × 3]	112 × 112	40	1
2	Fused_ MBConv1	112 × 112	16	2
3	Fused _ MBConv4	56 × 56	32	3
4	Fused_ MBConv4	28 × 28	48	3
5	MBConv4	14 × 14	96	4
6	MBConv6	14 × 14	112	6
7	MBConv6	7 × 7	192	9
8	Convolution layer [1 × 1], Pooling, and FC	7 × 7	1280	1

#### 3.2.2 The attention mechanism

During feature extraction, the CNN gathers a large amount of irrelevant background information and noise from the input image. This irrelevant information considerably influences the accurate identification of diseases. Using the attention mechanism the emphasis of the network is directed onto important feature information while suppressing noise and background, which significantly increases identification accuracy. The attention mechanism is a selective system that gives various feature information varied weights; for instance, it gives disease-specific information greater weight while giving background and noise less weight. Many studies have been conducted on attention processes, which are broadly classified as channel attention (CA) and spatial attention (SA) mechanisms (Guo et al., 2022). The SA mechanism performs well in probing the target’s position in the feature map, while the CA mechanism effectively searches for a specific target across several feature maps. Additionally, considering the combination of CA and SA modules in parallel or sequential, the sequential approach performs better in real-world application settings (Zhu et al., 2021; Guo et al., 2022).

**Figure 6:**
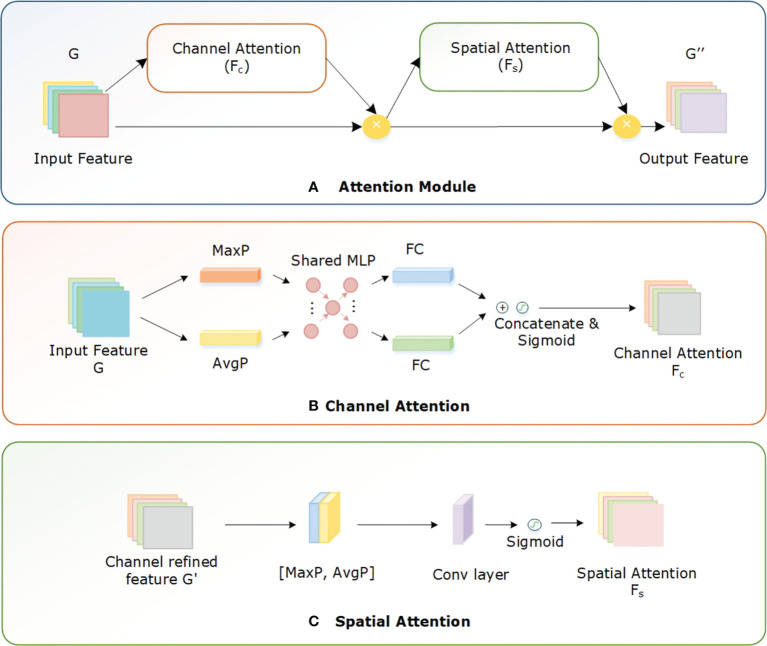
Structure of Attention module comprising channel and spatial attention.

In this study, we added the spatial-channel attention (SCA) module (Woo et al., 2018) to the Efficientnetv2 model so that the network can emphasize the specific information. As a result, the network learns to focus on disease-related important characteristics while ignoring irrelevant information acquired concurrently. Both the Efficientnetv2 CNN and SCA module produce an effective hybrid model, the CNN extracts high-level global information while SCA emphasizes specific features. During network training, the SCA module learns the relevance of interchannel correlations and spatial positions for the input features. Both the spatial and channel modules redistribute the weight of the characteristics in an adaptive manner after learning the essential information in both the channel and the spatial dimensions. **Figure 6** shows the structure of the SCA block.

Assume *G ⋲ Y^C×W×H^* is an intermediate keypoint map with dimensions *C×W×H* from the Efficientnetv2 CNN model is passed to the SCA module. The CA block generates a 1D channel feature map *F_C_ ∈ Y^1×1×C^*, whereas the SA block produces a 2D spatial feature map *F_s_ ∈ Y^1×W×H^*. The entire function of the SCA module is given by:


(1) $$\text{G}=\text{CA }\left(\text{G}\right)+\text{SA }\left(\text{G}\right)={\text{F}}_{\text{c}}\;\left(\text{G}\right)*\text{G }+\text{G}*{\text{F}}_{\text{s}}\left(\text{G}\right)$$

where * denotes the dot product of elements. To compute the input keypoints *G*, both CA and SA modules use maximum pooling (MaxP) and average pooling (AvgP) layers. In the CA block, the result of these two pooling operations are added together to produce the final keypoint map. The CA is computed as:


(2) $${\text{F}}_{\text{c}}\left(\text{G}\right)\;=\text{σ }\left(\text{Mlp }\left(\text{MaxP }\left(\text{G}\right)\;\right)+\text{Mlp }\left(\text{AvgP }\left(\text{G}\right)\right)\right)$$

(3) $$=\sigma \left({\text{X}}_{1}\left({\text{X}}_{0}\left({\text{G}}_{\text{max}}^{\text{c}}\right)\right)+{\text{X}}_{1}\left({\text{X}}_{0}\left({\text{G}}_{\text{avg}}^{\text{c}}\right)\right)\right)$$

where *σ* shows the sigmoid activation method, the *X_1_* and *X_0_* are learning weights, the ${\text{G}}_{\text{max}}^{\text{c}}$ and ${\text{G}}_{\text{avg}}^{\text{c}}$ are average-pooled and max-pooled features, and MLP is a multilayer perceptron. The SA block generates the spatial attention map by concatenating the final feature acquired from channel attention. These are the values of ${\text{G}}_{\text{avg}}^{s}$ and ${\text{G}}_{\text{max}}^{\text{s}}$ along the channel, dimension to emphasize the regions carrying important information. These values are combined, and a convolutional layer is used to execute the convolution operation. SA is determined by:


(4) $${\text{A}}_{\text{s}}\left(\text{G}\right)=\sigma \left({\text{f}}^{7\times 7}\left(\left[\text{AvgP}\left(\text{G}\right);\;\text{MaxP}\left(\text{G}\right)\right]\right)\right)$$

(5) $${\text{A}}_{\text{s}}\left(\text{G}\right)=\sigma \left(f^{7\times 7}\left(\left[G_{\text{avg}}^{\text{s}};G_{\text{max}}^{\text{s}}\right]\right)\right)$$

Where *f* denotes the convolutional operation with a kernel of the size 7×7.

### 3.3 Loss function

A loss function is utilized to measure how well the model predicts the data during training. The recognition of maize diseases is a multi-class categorization problem. Typically, multi-class classification problems employ the categorical cross-entropy (CCE) loss function. The main drawback of CCE loss is that it presumes equal learning across all categories (Sambasivam and Opiyo, 2021). In class-imbalanced training, this negatively impacts the training and classification performance. In order to focus on learning the minority classes, a focal loss is introduced, which modifies the conventional CCE loss function by down-weighting the majority class (Lin et al., 2017; Tran et al., 2019). We used the multi-class focal loss function during the training phase to compensate for class imbalanced data and improve the model classification accuracy. The categorical focus loss in a setting with multi-class maize disease classification is defined as:


(6) $$\text{Loss}\left(\text{k},\text{y}\right)=-\sum_{k=1}^{c}\eta_{k}{\left(1-y_{k}\right)}^{\gamma }\;\text{log}(y_{k})$$

where *y* and *k* are the expected probability distribution and the total number of classes, respectively. The hyper-parameter *η* and *γ* are the weighting factor and modulating parameter set as 1, respectively.

## 4 Experiment and results

This section includes a description of the database utilized to assess the performance of the proposed EANet model. It also describes the implementation and different experiments performed for the evaluation. A thorough investigation of the obtained results after executing various experiments is presented.

### 4.1 Implementation details

The described approach was developed using Python with Tensorflow, and Keras DL framework. The training and testing of the models were executed in the Google Colaboratory (Colab) setting. In the introduced method, transfer learning was employed to train the models on the dataset for maize leaf disease classification. Transfer learning is employed to improve the efficiency of feature learning and the generalization of the proposed method. The weight parameters of the EffectiveNetV2 model trained on ImageNet (Huang et al., 2017) were used to initialize the training. The model was fine-tuned by using the maize disease dataset to learn the disease features from the input samples during training. As a result, the weight values in the layers were updated. Fine-tuning a network with transfer learning is usually much faster and easier than training a network with randomly initialized weights of the network from scratch. **Table 3** presents the details of network training parameters. The learning rate was set as 0.001. It was set to automatically decline by 0.1 after every 4 epochs for a total of 25 epochs, with no improvement in validation loss. After 10 epochs of no progress, the early stopping strategy was utilized to halt the model training in order to prevent overfitting. We partitioned the input dataset into 7:3 train and test sets. To train the model and assess over-fitting, we further divided the training set 9:1 into training and validation sets. The test set was used to assess the effectiveness of the model.

**Table 3:** Model training parameters.

Parameter	Value
Epoch	25
Learning-rate	0.001
Batch-size	16
Optimization function	Stochastic gradient descent

### 4.2 Evaluation parameters

In this study, we employed precision(P), recall(R), accuracy(Acc), the F1-score (FS) and G-mean (GM) metrics to evaluate the model effectiveness in identifying maize leaf diseases. The following are the formulae for these measuring indicators:


(7) $$P=\frac{T_{p}}{T_{p}+F_{p}}$$

(8) $$R=\frac{T_{p}}{T_{p}+F_{n}}$$

(9) $$Acc=\frac{T_{p}+T_{n}}{T_{p}+T_{n}+F_{p}+F_{n}}$$

(10) $$FS=\frac{P*R}{P+R}\times 2$$

(11) $$GM=\sqrt{R*\frac{T_{n}}{T_{n}+F_{p}}}$$

where the true-positive, *T_P_*, represents the number of images that were classified as correctly diseased class i.e. GLS, CR, NLB, SLB, and healthy. Whereas false-positive, *F_P_*, represents the images classified incorrectly as diseased and in reality they are healthy. Moreover, false-negative, *F_n_*, represents the samples that are classified as healthy and belong to the diseased class. True-negative, *T_n_*, are those images that are classified as diseased, and in reality, they belong to the diseased class.

### 4.3 Evaluation of proposed model

In this sub-section, we presented the classification results of the EANet model for maize leaf disease obtained using images from the test set. To evaluate the classification performance, we computed the Acc, P, R, FS, and GM values for each class separately. The quantitative assessment results of the EANet model on the test set are given in **Table 4**. From **Table 4**, it can be seen that the EANet model shows remarkable performance in identifying multiple maize leaf diseases. The results show that the majority of samples in each category were accurately identified. The higher values of P, R, FS, and GM indicate the better class-wise prediction ability of the model on the employed database. More specifically, the recognition ability of our approach in terms of average P, R, FS, and GM values achieved is 98.90%, 98.87%, 98.89%, and 98.89%. These results show the overall effectiveness of the EANet model in the classification of healthy and multiple types of disease-affected maize leaves captured under various challenging environmental conditions.

**Table 4:** The class-wise quantitative assessment outcomes of the EANet model.

Category	P (%)	R (%)	FS (%)	GM (%)
Healthy	98.41	97.83	98.13	98.18
Grey Leaf Spot	98.76	98.69	98.72	98.65
Common Rust	100	100	100	100
Northern leaf Blight	98.67	99.03	98.85	98.93
Southern leaf blight	98.51	98.93	98.77	98.71

**Figure 7:**
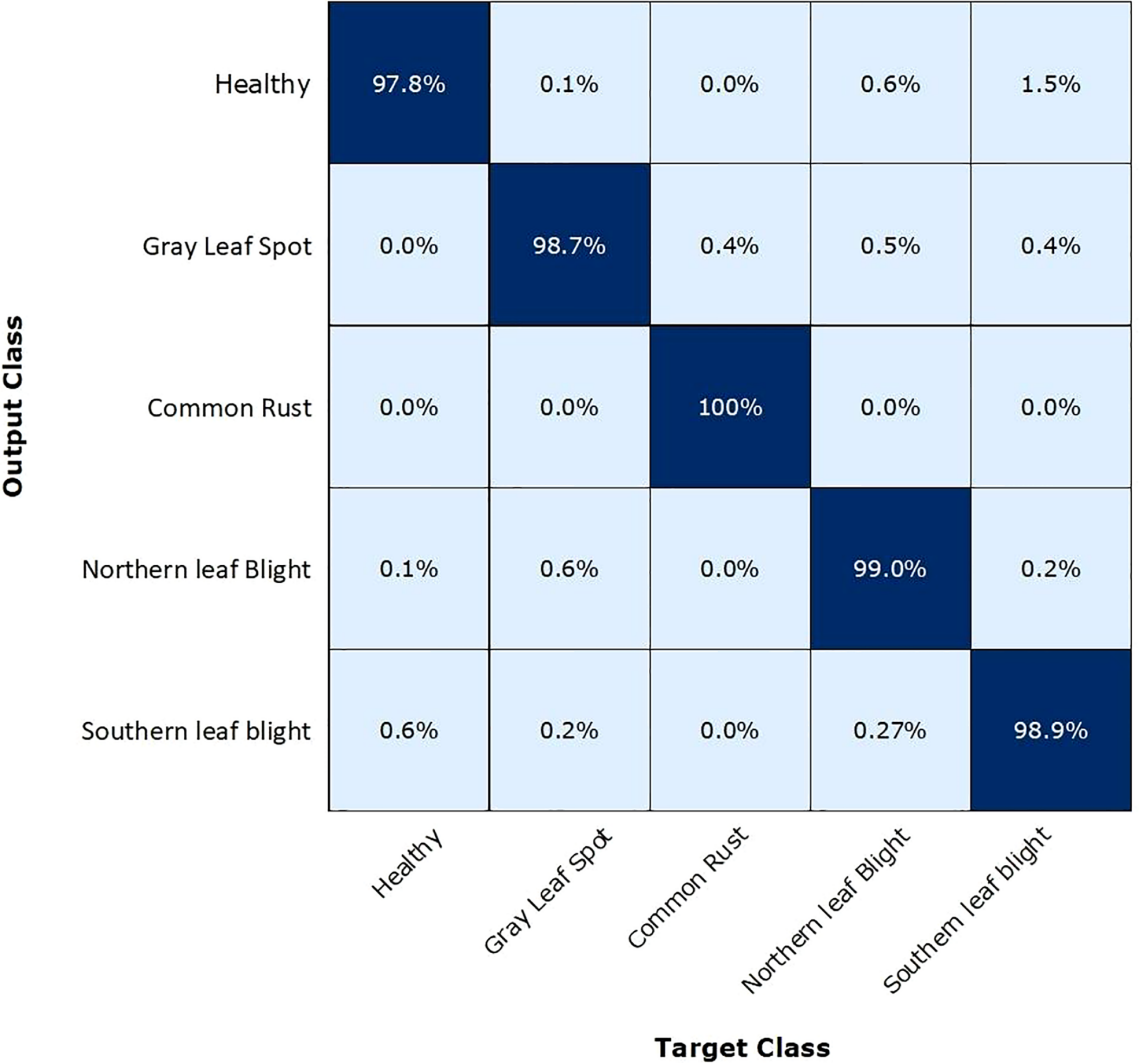
Confusion matrix of classification results for maize leaf disease using the test set.

**Figure 7** presents the confusion matrix (CM) summarizing the categorization accuracy of the EANet model. The percentage of trained model predictions that matched the class levels of test data accurately is represented by the diagonal matrix values, whereas the off-diagonal elements correspond to inaccurate predictions. The values shown in **Figure 7** depict that we have attained the highest true-positive rate for the CR class with a score of 100%. While we have attained the lowest true-positive rate for the healthy class with a score of 97.80%. The other classes such as NLB, GLS, and SLB have achieved a true-positive rate of 99%, 98.7%, and 98.9%, respectively. Overall, we can say that our approach is proficient in recognizing the CR class, while it has shown a few misclassifications in predicting the images of the healthy and other classes. The reason for the inaccuracy may be mainly caused due to the similarity of the visual symptoms of healthy samples with NLB, GLS, and SLB categories. Moreover, the interference of background also leads to the incorrect identification of these samples. Hence, it can be said that for all the classes, we have attained notable results with the EANet model in a real-world environment setting.

**Figure 8:**
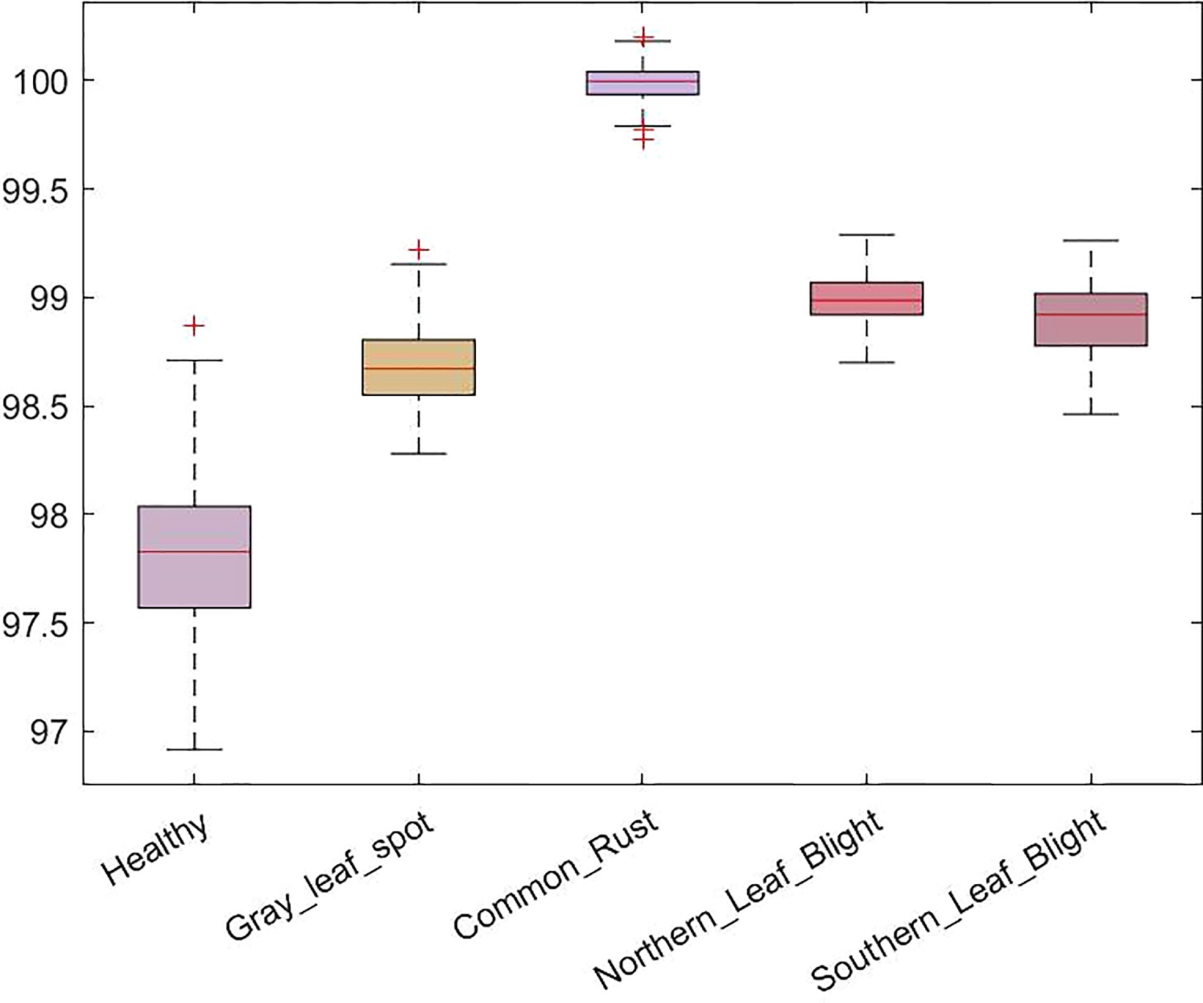
Box-plot of the accuracy for the proposed method on the test set.

We have also reported the accuracies of five maize leaf disease classes in a boxplot in **Figure 8**. The boxplot indicates the distribution of classification accuracy over different classes. According to **Figure 8**, our method attained the average accuracy values of 97.8%, 98.7%, 100%, 99%, and 98.9% for maize leaf disease classes i.e. healthy, GLS, CR, NLB, and SLB respectively. More specifically, we obtained an average classification accuracy of 98.94% with a low error rate on all classes that exhibit the efficacy of the proposed approach. The presented results show that our approach is robust against variations in disease appearance and can accurately identify the disease in presence of a complex background environment. The reason for the improved maize leaf disease classification performance is the correctness of the employed keypoint computation technique paired with the spatial-channel attention that represents each maize leaf disease class in a discriminative manner using inter-channel connection and space-wise point characteristics.

**Figure 9:**
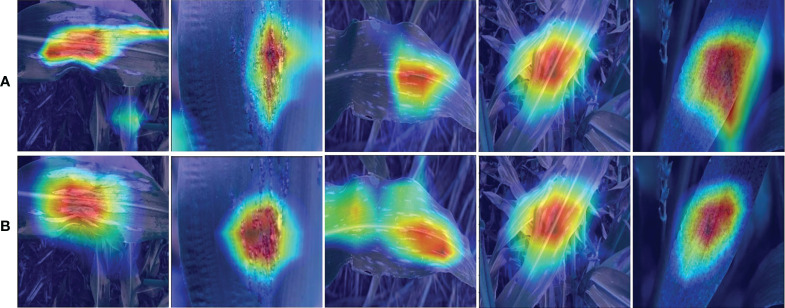
Sample attention heatmaps of the proposed approach for the categorization of maize diseases, **(A)** without attention mechanism and **(B)** with attention mechanism.

The infected region of the image provides critical information for disease identification when diagnosing maize leaf diseases using an automatic identification approach, however, the background region of the image frequently interferes. We examined the proposed model using Grad-CAM to assess which areas of the input images were useful for the network categorization results and the findings are presented in **Figure 9**. From **Figure 9**, it can be seen that the EANet model has learned to focus on relevant visual aspects and the results are based on reasonable attributes. Based on this observation, we may infer that while recognizing maize leaf disease the Efficientnetv2 model computes discriminative features, and the SCA module assists in determining the position of important information and enhancing information expression in key regions, hence improving specific disease recognition. The heat map analysis experiment demonstrated the capability to identify maize leaf diseases from a visual perspective.

### 4.4 Comparative analysis with different DL networks

Deep features are effective for image recognition tasks. We performed an experiment to compare the feature learning ability of various DL models using the maize leaf disease database. For this reason, we considered eight other commonly used CNN models such as Alexnet (Krizhevsky et al., 2012), GoogleNet (Szegedy et al., 2015), VGGNet (Simonyan and Zisserman, 2014), ResNet50 (He et al., 2016), InceptionV3 (Szegedy et al., 2016), and DenseNet-201 (Huang et al., 2017). EfficientNetv1 (Atila et al., 2021) and EfficientNetv2 (Tan and Le, 2021). These networks were trained using the transfer-learning approach, and the weights pre-trained on ImageNet (Deng et al., 2009) were used to initialize the network parameters. The classification layer was altered with a new softmax layer having the number of output classes in our database. We analyzed the acquired classification results of these models on train and test sets of the maize leaf disease database. We also assessed their computational complexity in terms of network parameters and the sample processing time to compare their performance with proposed approach.

**Table 5** shows the comparative results obtained by the proposed and other DL approaches. To compare DL models, we first analyzed model complexity in terms of trainable network parameters and sample processing time required. The **Table 5** shows that the proposed EANet model has fewer training parameters and requires less processing time to categorize the various maize leaf diseases than the peer techniques. The VGG-16 model contains the most network parameters i.e., 143M, while the DenseNet model is the most expensive in terms of processing time. In comparison, the proposed EANet model has 8.23 million parameters, which is fewer than all other models and requires a less processing time of 1,051 seconds, demonstrating the efficacy of the proposed approach. The addition of the attention module to EfficientNetV2 slightly increased the number of parameters while considerably improving classification results. **Table 5** illustrates that the suggested method offers a lightweight approach for maize leaf disease categorization as compared to other DL models.

**Table 5:** Comparative results of the proposed technique with other DL models.

Models	Training accuracy (%)	Testaccuracy (%)	Total trainable Parameter (M)	Processing Time (s)
AlexNet	90.46	87.12	62.3	1109
VGG-19	92.52	90.34	143	1007
Inception V3	93.67	92.82	23.8	3216
ResNet50	89.78	87.58	23.72	2536
DenseNet-201	98.31	94.17	20	3446
MobileNetv2	92.49	84.38	4.32	1013
EfficientNet	97.17	93.52	9.1	1236
EfficientNetv2	97.91	94.37	8.1	1083
Proposed model (EANet)	99.89	98.94	8.23	1051

**Figure 10:**
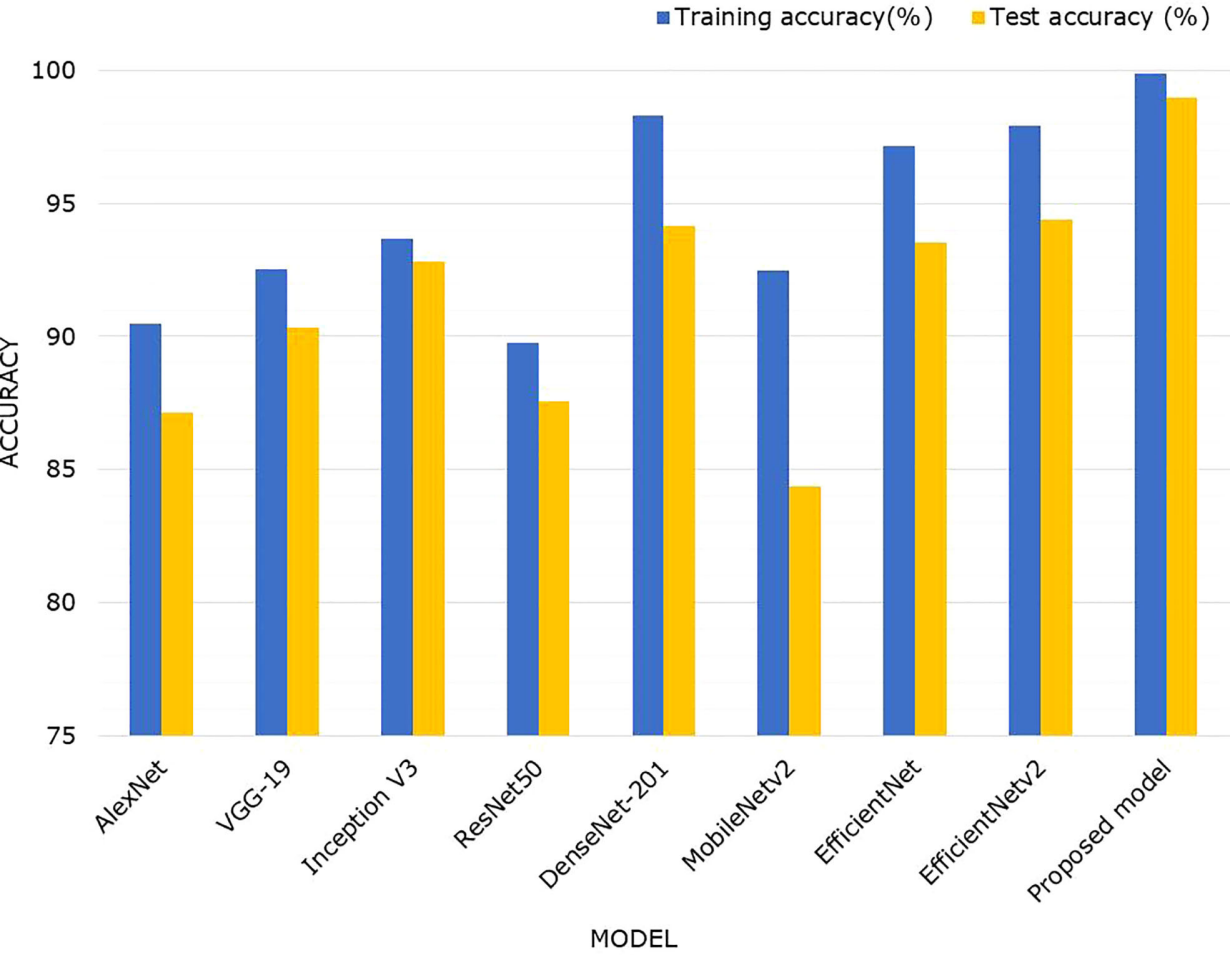
Performance comparison of the proposed technique with other CNN models.

**Figure 10** depicts a comparison of classification accuracies using a bar graph to better summarize the findings. It clearly shows that, when compared to AlexNet, VGGNet, InceptionV3, ResNet50, MobileNetv2, EfficientNetv1, and EfficientNetv2, the proposed model outperformed in identifying maize leaf diseases. More specifically, the suggested EANet model had an overall test accuracy of 98.94% for the maize disease classification, which was 11.82%, 8.6%, 6.12%, 11.36%, 4.77%, 14.56%, and 5.42%, 4.57% higher than AlexNet, VGGNet, InceptionV3, ResNet50, DenseNet-201, MobileNetv2, EfficientNetv1, and EfficientNetv2 model, respectively. By comparing the proposed model with its peer model such as EfficientNetv2, we observed that adding a spatial-channel attention mechanism to the EfficientNetv2 model significantly improves its ability to recognize maize disease. Without the attention mechanism, the EfficientNetv2 model accuracy for maize disease identification in the testing dataset was 94.37%, whereas the proposed EANet model attained an average performance increase of 4.57%.

In summary, after a thorough evaluation of existing DL models on the maize disease database, we observed that the proposed EANet model can precisely recognize multiple maize leaf diseases in field conditions. For the majority of assessment metrics, the EANet model outperforms other DL models utilized in the comparison study. The reason for the better performance of the proposed method is its improved network design, which extracts discriminative keypoints by focusing on disease spots rather than background noise information, thereby improving the classification accuracy.

### 4.5 Comparative analysis with other state-of-the-art models

To further assess the proposed EANet model performance, a comparison with current state-of-the-art maize disease identification methods is performed. The comparative findings are shown in **Table 6**.

**Table 6:** Comparison of the proposed technique with other state-of-the-art.

Technique	Data source	Diseases	Accuracy (%)
EfficientNet-B0 (Liu et al., 2020)	Crop disease AI Challenge dataset	Eight	98.52
Deep feature fusion using EfficientNetB0, and DenseNet121 (Amin et al., 2022)	PlantVillage	Healthy, GLS, CR, and NLB	98.56
CNN with multi-activation function (Zhang et al., 2021)	Private	Healthy, sheath blight, CR, NLB	97.41
Dense dilated convolutional blocks with attention fusion (Zeng et al., 2022a)	Private	Healthy, CR, NLB, gnawing beet armyworm, downy mildew, and GLS	95.4
Improved Googlenet with dilated inception module and channel attention (Yin et al., 2022)	Private	SLB	97.12
DenseNet with depth depthwise separable convolution and attention block (Chen et al., 2021)	PlantVillage and private	Healthy leaf images, GLS, CR, and NLB	95.86
Vision transformer with multi-head attention (Qian et al., 2022)	PlantVillage and private	Healthy, GLS, southern corn rust and SLB	98.7
**Proposed (Efficientnetv2 with SCA module)**	PlantVillage, Dataset provided by website (Ahmad et al., 2021) and (Qian et al., 2022)	Healthy, CR, GLS, SLB, NLB	98.94

As demonstrated in **Table 6**, when EANet is compared to other methods described in the literature, it has a significant improvement in performance. More specifically, the proposed framework attained an average accuracy value of 99.98% which is higher than other comparative methods. The studies (Liu et al., 2020; Zhang et al., 2021; Amin et al., 2022) used various deep CNN architectures and the PlantVillage maize disease dataset as transfer learning. Few of them employed the attention method in CNNs to enhance classification accuracy (Chen et al., 2021; Zeng et al., 2022a; Qian et al., 2022; Yin et al., 2022). However, the accurate identification of maize disease is difficult under realistic field settings. The studies may (Ahmad et al., 2021; Chouhan et al., 2021; Xiang et al., 2021; Corn or maize leaf disease dataset, 2022; Yin et al., 2022) suffer from the model over-fitting issue as a result of their complex network structures. Moreover, the comparative approaches show robust performance on samples having a simple background or limited disease categories. In comparison to these methods, the proposed EANet model employs an Efficientnetv2 network paired with a spatial-channel attention mechanism which not only assists in computing important image features but also decreases model training complexity while also providing a computational benefit. The proposed approach is evaluated on a database containing samples having heterogeneous field environments such as background noise and inconsistent lighting strengths. Thus, has the capacity to recognize healthy and different maize leaf diseases such as GLS, NLB, CR, and SLB under complex background settings.

## 5 Conclusion

In this work, we presented an automated approach for classifying maize diseases using DL. We proposed EANet, an Efficientnetv2 CNN model coupled with an attention mechanism to identify maize disease, which has a relatively small model size and good accuracy. The introduced architecture with spatial-channel attention enhances the capability of feature learning of the model from raw images captured in real-environment settings such as the complex background and varying lightning. An impressive performance is attained on test images by conducting a series of different experiments. The proposed method attains an overall training and testing accuracy of 99.89% and 98.94%, respectively for recognizing the five major maize leaf disease classes. The results show that the proposed method can effectively categorize maize leaf disease in the presence of complex background settings and various distortions, such as varying brightness, contrast, color, position, angle, and structure. In all evaluation metrics, the presented model outperforms the other CNN models utilized in the comparison experiments. Furthermore, the findings of the visual analysis experiments also indicate that the suggested technique developed in this work can not only properly identify infected regions but also sufficiently transmit information about such areas while recognizing the specific disease. In the future, we intend to use the model on portable devices for the purpose of real-time monitoring and identifying maize diseases. Furthermore, we also plan to make it more useful for real-world applications to classify other maize leaf diseases as well.

## Data availability statement

The original contributions presented in the study are included in the article/supplementary material. Further inquiries can be directed to the corresponding author.

## Author contributions

SA: Conceptualization, Methodology, Validation, Writing-Original draft preparation, Supervision; MM: Data curation, Methodology, Software, Validation, Writing-Reviewing and Editing. All authors contributed to the article and approved the submitted version.

## Acknowledgments

The researchers would like to thank the Deanship of Scientific Research, Qassim University for funding the publication of this project.

## Conflict of interest

The authors declare that the research was conducted in the absence of any commercial or financial relationships that could be construed as a potential conflict of interest.

## Publisher’s note

All claims expressed in this article are solely those of the authors and do not necessarily represent those of their affiliated organizations, or those of the publisher, the editors and the reviewers. Any product that may be evaluated in this article, or claim that may be made by its manufacturer, is not guaranteed or endorsed by the publisher.
